# Current practice and barriers for transition of care (TOC) in pediatric surgery: perspectives of adult surgeons from different subspecialties

**DOI:** 10.1007/s00383-025-05969-0

**Published:** 2025-01-28

**Authors:** Woon Teen Sia, Jia Chyi Tay, Tiong Chan Lee, Shireen Anne Nah, Mohan Arunasalam A. Nallusamy, Hans Alexander Mahendran

**Affiliations:** 1 Monash Medical University, Clinical School Johor Bahru No. 8, Masjid Sultan Abu Bakar, 80100 Johor Bahru, Johor Malaysia; 2 Department of Surgery, Sultanah Aminah Hospital, Jalan Persiaran Abu Bakar Sultan, 80100 Johor Bahru, Johor, Malaysia; 3 Division of Paediatric and Neonatal Surgery, Department of Surgery, Faculty of Medicine, University of Malaya, 50603 Kuala Lumpur, Wilayah Persekutuan Kuala Lumpur, Malaysia; 4 Department of Surgery, Sultanah Bahiyah Hospital, Km 6, Jln Langgar, 05460 Bandar Alor Setar, Kedah Malaysia

**Keywords:** Follow-up, Long-term care, Surveillance, Congenital abnormalities

## Abstract

**Introduction:**

The advancements in neonatal resuscitation and surgical care have enabled children with congenital abnormalities to receive corrective surgeries and achieve lifespans well into adulthood. These patients may require long-term follow-up as they continue to have risks of developing sequelae from their original diseases or surgical interventions.

**Purpose:**

This study aimed to investigate the current practice and barriers to the transition of care (TOC) from the perspectives of adult surgeons.

**Methods:**

A cross-sectional study was conducted with purposive sampling of adult surgeons from different subspecialties. An online self-administered questionnaire was distributed.

**Results:**

There were 60 respondents. 62% of the respondents had experience managing referrals for continuation of care or complications in patients with congenital abnormalities. 38% of the respondents believed that TOC should be implemented when patients reached ages 17–18 years. 93% of the respondents agreed that a proper TOC model would greatly benefit patient care, and 97% asserted the need to develop TOC pathways in Malaysia. The absence of a proper guideline was the greatest barrier to a smooth TOC.

**Conclusion:**

This study offered insights into the obstacles to TOC based on the perspectives and experiences of adult surgeons who participated. While not exhaustive, our study provided a better understanding of the challenges in developing the appropriate referral pathways for the continued care of these patients.

**Supplementary Information:**

The online version contains supplementary material available at 10.1007/s00383-025-05969-0.

## Introduction

The transition of care (TOC) from pediatric to adult care providers is carefully defined as ‘a purposeful, planned process that addresses the medical, psychosocial, and educational/vocational needs of adolescents and young adults who have had prior congenital pathologies as they move from pediatric/child-centered to an adult-oriented healthcare system’ [1, 2]. The advancement of neonatal resuscitation, neonatal intensive care, and surgical expertise has now enabled children with congenital abnormalities to achieve lifespans well into adulthood [3]. However, the nature of their illness requires long-term follow-up as they continue to have risks of developing sequelae throughout their lives. Recent studies reveal that most surgical conditions could significantly reduce patient quality of life in the long term [4]. The potential morbidities and mortality trigger the need for a structured TOC for these patients.

The Royal College of Surgeons of England Children’s Surgical Forum acknowledged transitional care as “a process, not a single event” where every National Health Service (NHS) trust should have a policy and an identified lead. Despite international recognition and the urgency for a better framework, few healthcare systems and pediatric centers have practiced the TOC model due to the challenges of lacking human resources, funding, and facilities [5].

Currently, no clear guideline or policy is addressing the TOC for adolescents with complex surgical conditions that manifest in childhood in Malaysia. A locally conducted survey revealed that most of the pediatric surgeons managed their patients well into adulthood. This is less than ideal, and the study identified the need to develop protocols ideally within the next 3–5 years [6]. This study, however, only focused on the current practices and perspectives of pediatric surgeons in Malaysia. The perspectives of adult surgeons from different subspecialties should be investigated to better understand how to bridge the divide. The identification of barriers and gaps would assist in developing appropriate referral pathways for the continued care of these patients. Thus, our study aimed to identify possible barriers in the TOC of pediatric surgical patients to adult care and to help develop a proper referral pathway.

## Methods

The study was performed between December 2023 and March 2024. This was a cross-sectional quantitative study involving an online self-administered questionnaire that was distributed among qualified surgeons managing adult patients in Malaysia. All respondents were welcomed regardless of years of experience and institutions. Purposeful sampling was performed to target surgeons from the subspecialties such as breast and endocrine surgery, colorectal surgery, general surgery, hepatopancreaticobiliary surgery, upper gastrointestinal surgery, and vascular surgery.

There were no existing validated questionnaires about this subject. Thus, the questionnaire was developed in stages. At the initial stage, a literature review of TOC was performed by the researchers to develop the preliminary questionnaire (1st draft). Discussions were held among authors, which included both pediatric and adult surgeons. Valuable opinions were received from the authors of a Malaysia-based study, “Transition of Care in Paediatric Surgery: Current Practices and Perspectives of Pediatric Surgeons in Malaysia” [6]. Then, the draft was sent to a focus group composed of 7 consultant surgeons from different subspecialties to obtain expert opinions for the face and content validity (2nd draft). This focus group consisted of a plastic surgeon, a hepatobiliary surgeon, a neurosurgeon, a colorectal surgeon, and 3 upper gastrointestinal surgeons. They were experts in their respective fields of surgery and had more than 10 years of service in government and private healthcare settings in Malaysia.

The questionnaire was finalized after this validation process. The final questionnaire consisted of three parts. The first part identified the respondents according to specialty, current position, years of experience, and institution. The second part identified current practices and barriers experienced by surgeons. The third part determined the perspectives of surgeons in developing a proper TOC pathway.

The questions were designed to require a “yes” or “no” answer. Surgeons were required to choose or list the factors that necessitated the TOC based on their experiences (in the second part) based on their perception.

The third part involved responses that utilized a Likert Scale of Agreement (1—strongly disagree; 2—disagree; 3—neither agree nor disagree; 4—agree; 5—strongly agree) to evaluate surgeons’ responses to some statements and the perceived barriers to a smooth TOC. There was a segment for respondents to share their experiences and opinions in the final part of the questionnaire.

Statistical analysis was performed using the Statistical Package for the Social Sciences (SPSS), Version 26.0 (IBM Corp., Armonk, New York, USA). Descriptive analysis was performed. Categorical data were presented as frequency and percentage, while numerical data were presented as mean and standard deviation or median and interquartile range. Graphical representations of data (Likert scale) were included for better visualization.

The survey was disseminated via email to surgeons who fulfilled the inclusion criteria: must be a surgeon and practicing in a university hospital or Ministry of Health (MOH). Surgeons were identified through professional networks and directories to ensure eligibility. In total, 90 eligible surgeons were identified and received the survey. 57 responses were needed to achieve a 95% confidence interval and 8% margin of error based on Yamane’s formula.

This study was approved by the Malaysia Research Ethics Committee (MREC), NMRR ID-23–02119-UPG (IIR). Informed consent was received from all respondents.

## Results

There was a total of 60 respondents who participated in the survey, with a response rate of 67%. The majority (67%) were general surgeons, followed by upper gastrointestinal surgeons (13%), hepatopancreaticobiliary and colorectal surgeons (8%, respectively), orthopedic, and vascular surgeons (2%, respectively). Among the respondents, there were 3 Heads of Services (HOS) in the Ministry of Health (MOH) and 13 Heads of Department in their respective institutions. 95% worked in government hospitals (42% in state, 30% in major specialist, 8% in minor specialist, 15% in districts), while 3% worked in university hospitals and 2% in private hospitals. The mean year of experience as practicing surgeons for the respondents was 7 years.

More than half (62%) had experience managing referrals from pediatric surgeons, and the majority had managed between 1 and 5 cases. 28% of the respondents reported the cases were co-managed by both adult and pediatric surgeons, 20% of the cases were solely managed by adult surgeons, 10% were managed by adult surgeons who had pediatric surgery experience, and the remaining 42% were solely managed by pediatric surgeons. Collaborations often involved co-managing the primary condition and surveillance for potential long-term complications.

**Fig. 1:**
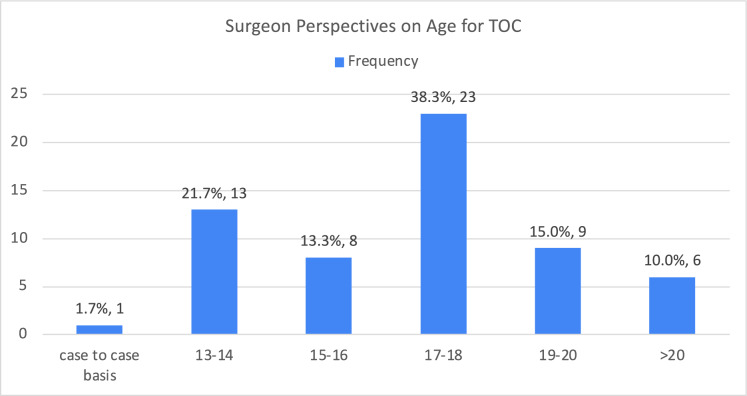
Surgeon perspectives on age for TOC

From the respondents’ perspectives, 17–18 years was the most appropriate age for TOC. The vast majority of the surgeons (63.3%) agreed that TOC should be started when patients are older than 17 years old (Fig. 1). All surgeons agreed that involvement of pediatric surgeons was essential for a successful and smooth TOC. Four-fifths of the respondents asserted that pediatric surgeons were needed to participate in patient care after the referral. The majority of the respondents (93%) agreed that TOC is beneficial for the continued care of these patients, and 97% of the respondents felt that TOC was needed in Malaysia.

**Fig. 2:**
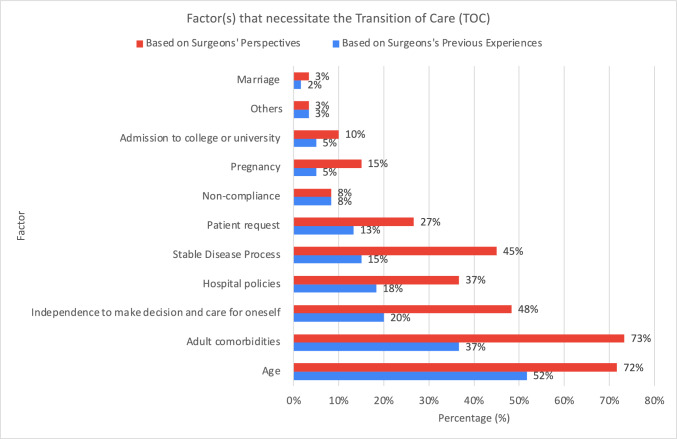
Factor(s) that necessitate the transition of care (TOC) based on surgeons’ previous experiences and perspectives

Among all the important factors that necessitated the TOC, increasing age, manifestation of adult comorbidities, and independence to make decisions and care for oneself remained the 3 major factors based on surgeons’ previous experiences and perspectives. This was followed by hospital policies, stable disease processes, patient requests, and non-compliance. Marriage, pregnancy, and admission to college or university appeared to be lower priorities from the perspectives of respondents (Fig. 2).

**Fig. 3:**
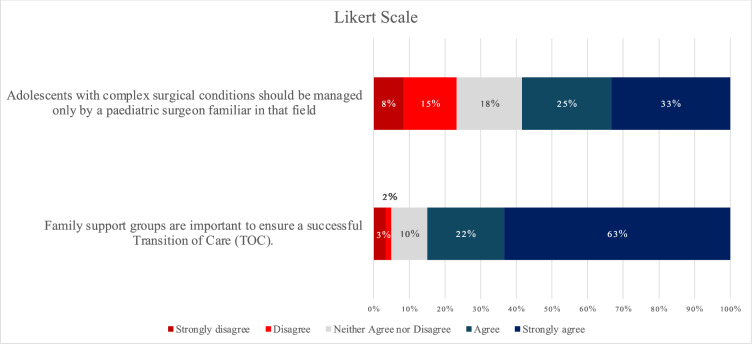
Likert scale of agreement

The degree of agreement among the respondents was further explored. A strong agreement was observed towards the need for family support to ensure a successful TOC (63% strongly agree, 22% agree). For patients with complex surgical conditions, 23% of the respondents believed that it was insufficient for the conditions to be managed solely by pediatric surgeons familiar with that field (Fig. 3).

**Fig. 4:**
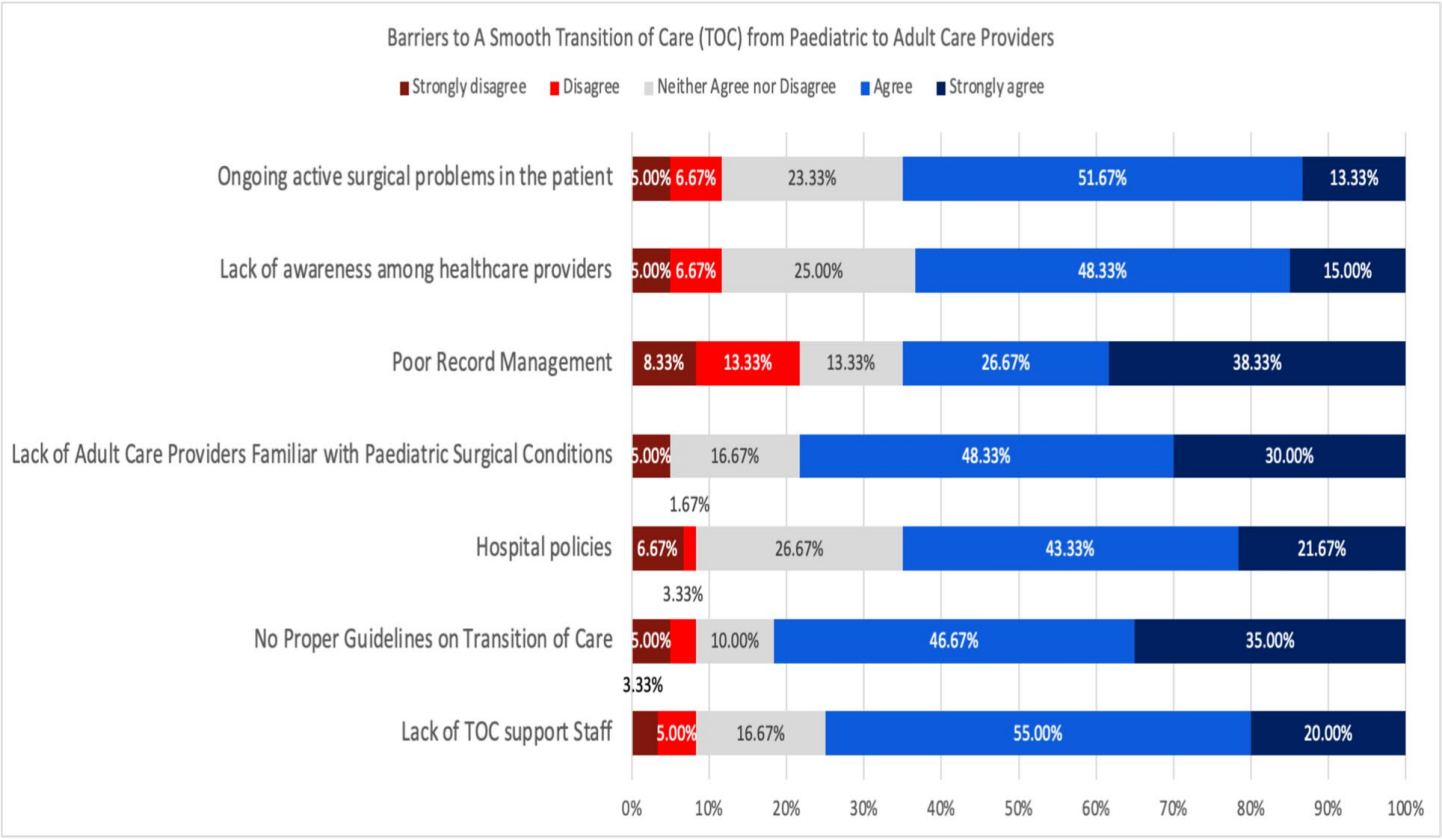
Barriers to a smooth transition of care (TOC) from paediatric to adult care providers

Regarding the barriers to a smooth TOC from pediatric to adult care providers, 81.67% of the respondents agreed that the absence of proper guidelines on TOC was the major barrier (20% strongly agree, 46.67% agree). This was followed by the lack of adult care providers familiar with pediatric surgical conditions (78.33%) and a lack of TOC support staff (75%). Poor record management and hospital policies were also in concern (38.33% and 21.67% strongly agree, respectively) (Fig. 4).

### Other barriers to TOC suggested by respondents

In the open comment section, other barriers suggested by respondents included:

- Fear of litigation or medicolegal implications for the management of complex conditions
- Patient’s psychological fear of transit to an unfamiliar adult care environment.
- Reluctance of pediatric care provider to release care of patients to the adult care provider.
- Reluctance of adult care providers to take over the care from a pediatric care provider.
- Limited knowledge of the latest advancements in disease management and hospital policies.
- Lack or poor communication among pediatric and adult surgeon.
- Lack of understanding/knowledge among adult surgeons in the management of congenital diseases.
- Lack of experienced adult care providers in managing patients with a background of congenital abnormalities.
- Lack of case-to-case management.
- Parents may benchmark the standard of care based on experience from the previous pediatric care provider.
- Movement of the specialists between hospitals.

## Discussion

The adult surgeon is one of the fundamental stakeholders in ensuring the smooth and successful TOC. The results of this study were important to reflect the current TOC practices and challenges experienced by adult surgeons in Malaysia. The exploration of barriers was helpful in driving the direction of model development and implementation. Significantly, there was a proven need for a structured and algorithmic TOC model. The low number of referred cases managed by adult surgeons (1–5 cases in our study) revealed that TOC was not practiced widely among adult surgeons, which was consistent with the previously mentioned study that focused on pediatric surgeons in Malaysia [6].

With the advancement of technologies and the improvement of healthcare quality, the number of patients who achieve longer lifespans is expected to expand in the coming years. Pediatric surgery is a relatively new specialty in Malaysia. Starting from 2010, pediatric surgery is no longer a subspecialty of general surgery, i.e., medical graduates who are ambitious to specialize in pediatric surgery may enter the specialization program without the prerequisite of being a qualified adult general surgeon. This direct path may be favorable to most aspiring pediatric surgeons, but the concern of pediatric surgeons having less experience in handling adult surgical problems persisted.

The key factors influencing TOC in our study included increasing age, adult comorbidities, and patient independence.

Age remained the main factor that necessitates the TOC based on surgeons’ experiences and opinions. Regarding one of the most commonly used TOC models—‘Ready, Steady, Go’, pediatric team would plan the transition in stages [2]. Young patients would start visiting the transition clinics, which consisted of both pediatric and adult teams, from 16 to 18 years old. This allowed formal communications and collaborations between surgeons and young patients might highlight their ongoing issues to the adult team [7]. Some studies suggested the start of the TOC in early adolescence [8]. From the respondents’ perspectives in our study, 17–18 years was deemed as the most appropriate age for the TOC. We acknowledged that the transition from pediatric to adult care was a gradual process that would occur at varying speeds depending on the conditions and individual circumstances. Our finding likely reflected the perspectives of adult general surgeons who participated in the study. However, evidence in the literature highlighted that transition planning should begin earlier, around the age of 12 years, to allow sufficient time for a gradual and effective transition process. This discrepancy might point to a gap in awareness among adult general surgeons about the challenges and nuances of transition, which were more commonly navigated by pediatricians and pediatric surgeons. In addition, this recommendation of age by adult surgeons in our study might be due to the local challenges, such as the shortage of experts in adolescent care and the lack of adolescent-friendly facilities [9].

The development of adult comorbidities was another paramount factor that demands TOC based on our study. The literature revealed that some patients moved on to lead a disease-free life post-surgery while some struggled with chronic symptoms, including physical and psychological disturbances, even after a successful surgery. Some disease-specific long-term morbidities included dysphagia in esophageal atresia, constipation in anorectal malformation and Hirschsprung’s disease, portal hypertension in biliary atresia, and pulmonary impairment in congenital diaphragmatic hernia [4]. There were also case series reporting the development of carcinomas in patients with a history of esophageal atresia and anorectal malformations in their 40s and 30s, respectively [10, 11]. This was concerning as regular follow-ups and disease surveillance might enable earlier detection and provide a better prognosis. Common adult comorbidities such as diabetes mellitus and hypertension were suggested to develop earlier in this patient population than in the general population [12, 13].

Almost all surgeons agreed that the TOC was beneficial to patient care and needed in Malaysia. This implied the support and interest of adult surgeons in improving the care of pediatric patients with complex surgical conditions. In addition, all surgeons claimed that the involvement of pediatric surgeons would lead to successful and smooth TOC. This aligned with the pediatric surgeons’ perspectives, which 84% of them expressed the obligation to provide consultations and care even after patients have been transferred to adult care [6].

In Malaysia, pediatric surgeons often continue to follow up with their patients into adulthood, as there is no formal handover process in place. Adopting and adapting TOC models from other countries might expedite the formation of an appropriate framework in Malaysia. The readiness of adolescents to navigate into the adult healthcare settings independently should be prioritized. The transitional care framework introduced by The North American Society for Paediatric Gastroenterology, Hepatology, and Nutrition (NASPGHAN), which eventually matched each adolescent with the adult care providers, might be studied and modified. This model ensured individualized and comprehensive care to patients [14].

Nevertheless, there were multiple barriers to TOC. In this study, adult surgeons perceived poor record management as the major barrier to a smooth TOC, followed by the absence of a proper guideline, lack of adult care providers familiar with pediatric surgical conditions, hospital policies, lack of TOC support staff, lack of awareness among healthcare providers, and ongoing active surgical problems in patients. By acknowledging and recognizing these barriers, they could be tackled accordingly.

There were some limitations to this study. The sample size of this study was small; it might not comprehensively represent adult surgeons from different subspecialties and different institutions. Besides, the responses were not stratified based on the specific congenital abnormalities managed by adult surgeons. Without this stratification, the specific referral pathway practiced by surgeons currently could not be identified and compared. Another limitation was that the questionnaire was not formally validated or assessed for internal reliability and consistency prior to its use. The robustness of the collected data and the generalizability of the findings might be reduced.

Following this study, which highlighted the need for a structured transition of care model in Malaysia, we recommended initiating deeper discussions between adult and pediatric surgeons. These discussions should aim to develop models such as combined clinics and multidisciplinary approaches to ensure a seamless TOC. A multidisciplinary team (MDT) might involve a core team (i.e., pediatric surgeons, adult surgeons, nursing, and imaging teams) and an extended team (i.e., anesthesiologist, psychologist/counselor, social workers, and nutritionist). This multidisciplinary approach serves to promote collaboration, create awareness among involved parties and bridge the gaps. Several issues that have to be addressed include the availability of resources (i.e., trained healthcare personnel, medical equipment, and country’s health expenditure), the acknowledgement of the need of TOC by each discipline involved, and the quantification of the need. The ideal model should be patient-centered, case-specific, and collaborative, ensuring that the unique needs of each patient are fulfilled.

## Conclusion

This study revealed that there is no predominant TOC guideline used in Malaysia, and many cases are only seen when emergency interventions are required. Most surgeons identified 17–18 years as the appropriate age for TOC, emphasizing the importance of individualized and gradual transition processes. Key factors driving TOC include age, adult comorbidities, and patient independences, while the involvement of pediatric surgeons is critical for successful TOC. Our study acknowledged the urgent need for a TOC framework to be implemented in Malaysia. Collaborative efforts among pediatric and adult surgeons were important to bridge the divide and ensure effective transitions. The major barriers must be addressed, including absence of guidelines, lack of experienced adult care providers and poor record management. Adopting multidisciplinary approaches and tailoring TOC models to local context would be constructive and practical. Future formal discussions and meetings should be organized between the pediatric and the adult teams to review the concept and approach of TOC in Malaysia. The findings of this study, along with previously published studies focusing on pediatric surgeons’ perspectives, provided valuable insights for developing patient-centered, collaborative, and comprehensive TOC guidelines.

## Supplementary Information

Below is the link to the electronic supplementary material.

Supplementary file1 (DOCX 19 KB)

## Data Availability

No datasets were generated or analyzed during the current study.
