# Supplementary material for: Current practice and barriers for transition of care (TOC) in pediatric surgery: perspectives of adult surgeons from different subspecialties
Source: Pediatr Surg Int. 2025 Jan 28;41(1):76. doi: 10.1007/s00383-025-05969-0 (PMC11774974; doi:10.1007/s00383-025-05969-0)
Supplement: Supplementary file 1 — Supplementary file1 (DOCX 19 KB) [file 383_2025_5969_MOESM1_ESM.docx]

Questionnaire:

**Part 1: Professional Profile**

- Division/ Specialty
  - General Surgery
  - Breast and Endocrine Surgery
  - Colorectal Surgery
  - Hepatopancreaticobiliary
  - Upper Gastrointestinal Surgery
  - Vascular Surgery
  - Cardiothoracic Surgery
  - Urology
  - Others
- Current position
  - Head of Department (HOD)
  - Head of Service (HOS)
  - Consultant
  - Specialist
  - Other
- Number of Years after completion of specialist degree
- Institution
  - Government: State Hospital/ District Hospital/ Major Specialist Hospital/ Minor Specialist Hospital
  - University Hospital
  - Private Hospital

**Part 2: “Do we have a problem”.**

- Experience of managing a patient with congenital abnormalities and sequelae
  - Yes🡪 Go to section **“Personal Experience in Transition of Care (TOC)”**
  - No🡪 Go to **Part 3: Adult Surgeons’ Perspectives on Transition of Care (TOC)**
- Section **“Personal Experience in Transition of Care”**
  - Based on personal experience, choose the factor(s) that necessitate the TOC of those patients:
    - Adult co-morbidities
    - Admission to College or University
    - Age
    - Hospital policies
    - Independent to Make Decision and Care for Oneself
    - Marriage
    - Non-compliance
    - Patient request
    - Pregnancy
    - Stable Disease Process
    - Other
  - How many cases of TOC have you managed?
    - 1-5
    - 6-10
    - 11-15
    - 16-20
    - >20
- Parties involved in the patient management.
  - Surgeon who perform adult surgery only
  - Surgeon who perform surgeries in adult and paediatric population
  - Both adult and paediatric surgeons 🡪 1 following question: “**Based on your experience, what is your perspective in regard to the cooperation between the adult and paediatric surgeons?”**
    - They co-manage the condition.
    - They manage based on anticipated complications.
    - Other.
  - Not sure
  - Others

**Part 3:** **Adult Surgeons’ Perspectives on Transition of Care (TOC)**

- In your opinion, what age should Transition of Care (TOC) of patients with congenital abnormalities to adult care begin?
  - 13-14
  - 15-16
  - 17-18
  - 19-20
  - >20
- In your opinion, who is (are) responsible for the successful and smooth Transition of Care (TOC)?
  - Paediatric surgeon
  - Adult surgeon
  - Parents
  - Patient
  - Other
- In your opinion, is paediatric surgeon required to take part in patient care after the referral/ transition?
  - Yes
  - No
- In your opinion, is personal communication with the adult healthcare providers necessary for the transition of the patient?
  - For every patient
  - For a selected group of patients.
  - For a patient with rare condition
  - Never
  - Other
- In your opinion, what are the major factor(s) that necessitates the Transition of Care (TOC)
  - Adult co-morbidities
  - Admission to College or University
  - Age
  - Hospital policies
  - Independent to Make Decision and Care for Oneself
  - Marriage
  - Non-compliance
  - Patient request
  - Pregnancy
  - Stable Disease Process
  - Other
- Do you agree that a proper Transition of Care (TOC) Model will bring great benefit towards the care of patient with congenital abnormalities?
  - Yes
  - No
- Do you agree that there is a need for a proper Transition of Care (TOC) model in Malaysia?
  - Yes
  - No
- Rating the following statement with Likert Scale of Agreement (1- Strongly Disagree; 2- Disagree; 3- Neither Agree nor Disagree; 4- Agree; 5- Strongly Agree)
  - Family support groups are important to ensure a successful Transition of Care (TOC).
  - Adolescents with complex surgical conditions should be managed only by a paediatric surgeon familiar in that field.
- Rate the Barriers to A Smooth Transition of Care (TOC) from Paediatric to Adult Care Providers with Likert Scale of Agreement. (1- Strongly Disagree; 2- Disagree; 3- Neither Agree nor Disagree; 4- Agree; 5- Strongly Agree)
  - Lack of Transition Care Support Staff
  - No Proper Guidelines on Transition of Care
  - Hospital Policies
  - Lack of Adult Care Providers Familial with Paediatric Surgical Conditions
  - Poor Record Management
  - Lack of Awareness among Healthcare Providers
  - Ongoing Active Surgical Problems in The Patient

Do you think that there is (are) other a barrier(s) to a smooth Transition of Care (TOC) of patients with congenital abnormalities to adult care? If yes, please kindly list down the barriers(s).
